# Comparative Study on the Oxidative Stability of Encapsulated Fish Oil by Monoaxial or Coaxial Electrospraying and Spray-Drying

**DOI:** 10.3390/antiox12020266

**Published:** 2023-01-24

**Authors:** Nor E. Rahmani-Manglano, Emilia M. Guadix, Charlotte Jacobsen, Pedro J. García-Moreno

**Affiliations:** 1 Department of Chemical Engineering, University of Granada, 18071 Granada, Spain; 2 National Food Institute, Technical University of Denmark, 2800 Kongens Lyngby, Denmark

**Keywords:** encapsulation, omega-3 polyunsaturated fatty acids, lipid oxidation, delivery systems, spray-drying, monoaxial electrospraying, coaxial electrospraying

## Abstract

The impact of the encapsulation technology on the oxidative stability of fish-oil-loaded capsules was investigated. The capsules (ca. 13 wt% oil load) were produced via monoaxial or coaxial electrospraying and spray-drying using low molecular weight carbohydrates as encapsulating agents (e.g., glucose syrup or maltodextrin). The use of spray-drying technology resulted in larger capsules with higher encapsulation efficiency (EE > 84%), whilst the use of electrospraying produced encapsulates in the sub-micron scale with poorer retention properties (EE < 72%). The coaxially electrosprayed capsules had the lowest EE values (EE = 53–59%), resulting in the lowest oxidative stability, although the lipid oxidation was significantly reduced by increasing the content of pullulan in the shell solution. The emulsion-based encapsulates (spray-dried and monoaxially electrosprayed capsules) presented high oxidative stability during storage, as confirmed by the low concentration of selected volatiles (e.g., (E,E)-2,4-heptadienal). Nonetheless, the monoaxially electrosprayed capsules were the most oxidized after production due to the emulsification process and the longer processing time.

## 1. Introduction

Food fortification with omega-3 polyunsaturated fatty acids (PUFAs), especially EPA (C20:5 n-3) and DHA (C22:6 n-3), has become a focus of scientific research as a result of the recognized health benefits derived from their consumption [1]. However, their inclusion into complex food matrices still poses a challenge for the food industry due to their low oxidative stability, which eventually affects the organoleptic and nutritional properties of the final food product [2]. In this regard, the micro- and nanoencapsulation of oils rich in EPA and DHA (e.g., fish oil) to be used as omega-3 delivery systems stands out as the approach with the highest potential [3]. 

In oil-loaded capsules, the oil is trapped within a matrix composed of the encapsulating agent/s, which act as a physical barrier against prooxidants (e.g., oxygen) [4]. Furthermore, microencapsulated oil is more bioaccessible compared to neat oil, and depending on the properties of the emulsifying and encapsulating agent/s, its controlled or targeted release can be achieved [5].

Spray-drying is the preferred encapsulation technique in the food industry due to its multiple advantages, such as the high throughput and low cost [6]. Furthermore, the encapsulation of fish oil by spray-drying, as a source of omega-3 PUFAs, has been widely studied over the last decades [3]. However, this technology also presents some disadvantages. It has been demonstrated that fish oil emulsification and drying at high temperatures (160–200 °C) results in initial lipid oxidation [7]. Moreover, the large size range (~5–100 µm) and the broad particle size distribution of the resulting microcapsules may limit their use for certain food applications. Therefore, novel encapsulation technologies such as electrospraying have emerged as alternatives to conventional spray-drying. 

Electrospraying technology is based on applying a high-voltage electrostatic field between the end of an emitter and a grounded collector to cause the ejection of a solution [8]. When the electric force overcomes the surface tension of the solution droplet formed at the tip of the emitter, a charged jet is ejected in the direction of the collector. At sufficiently low viscoelasticity of the solution, the jet destabilizes, forming a spray of highly charged small droplets. These droplets self-disperse on their way to the collector due to electrostatic repulsion, avoiding particle agglomeration or coagulation [8]. One of the advantages of electrospraying over spray-drying is that heat is not required at any point in the process, since solvent evaporation occurs at ambient temperature when the droplets travel on their way to the collector. Moreover, contrary to the mechanically atomized droplets produced by spray-drying, the electrosprayed liquid droplets are smaller and show a monodisperse particle size distribution [9], which results in highly bioaccessible capsules with little organoleptic changes when incorporated into a food matrix. Nonetheless, it should be born in mind that smaller capsules may be more prone to oxidation due to an increased contact area between the lipids and prooxidants [10]. Fish-oil-loaded capsules have been produced by electrospraying in the monoaxial configuration with promising outcomes [8]. However, some authors reported initial lipid oxidation when water-soluble (bio)polymers were used as the encapsulating agent as a consequence of the emulsification process before drying [11,12].

Coaxial electrospraying modifies the monoaxial electrospraying process by introducing a coaxial emitter, which allows to electrospray two liquids simultaneously [13]. In brief, through the inner needle of the emitter flows the bioactive ingredient, and through the annular gap, between the inner and the outer needle, flows the (bio)polymer-based solution used as the encapsulating agent [3]. This technology is particularly interesting since it allows to produce capsules from two immiscible liquids without the need of producing first a dispersion or an emulsion of the bioactive compound. Thus, the initial fish oil oxidation caused by emulsification could be theoretically avoided by producing the capsules in the coaxial configuration. Furthermore, emulsion-based encapsulation methods (e.g., spray-drying) lead to a random distribution of oil droplets within the encapsulating matrix, favoring the presence of easily accessible surface oil. On the contrary, the use of optimum coaxial electrospraying could lead to a centralized distribution of the oil within the (bio)polymer-based wall, whose thickness can be easily controlled [13]. Therefore, capsules with a higher load capacity and encapsulation efficiency (EE) might be produced [3]. Despite the potential advantages of coaxial electrospraying over its monoaxial counterpart, only one study has been reported in the literature on the encapsulation of the short-chain omega-3 fatty acid α-linolenic acid (ALA) using proteins as encapsulating agents (e.g., zein or gelatin) [14]. These authors observed that coaxial electrospraying resulted in better entrapment of the core material within the encapsulating matrix compared to monoaxial electrospraying. However, the oxidative stability of the resulting coaxial encapsulates was only improved when gelatin was used as the outer encapsulating agent, and not zein, which does not correlate with the EE values reported.

Low molecular weight (LMW) carbohydrates such as glucose syrup (GS) and maltodextrin (MD) have been extensively used for the encapsulation of fish oil by spray-drying [3], and their use has also been reported in the encapsulation of omega-3 PUFAs using monoaxial electrospraying technology [11,12]. Nonetheless, to the best of the authors’ knowledge, fish-oil-loaded capsules have not yet been produced by coaxial electrospraying using GS or MD as the main encapsulating agent. In addition, a systematic study about the impact of the encapsulation technology on the oxidative stability of fish-oil-loaded capsules has not yet been reported. 

Taken altogether, the objective of this work was to investigate the production of fish-oil-loaded capsules using different encapsulation technologies such as spray-drying and monoaxial or coaxial electrospraying using GS or MD as the main encapsulating agent. Whey protein concentrate hydrolysate (WPCH), with high emulsifying and antioxidant activity [15,16], was used for the first time as the emulsifier in the production of fish-oil-loaded capsules via monoaxial electrospraying. For coaxial electrospraying, neat fish oil was infused as the core solution. The resulting encapsulates were characterized in terms of their morphology, particle size distribution, and encapsulation efficiency, and their oxidative stability was monitored over six weeks of storage at ambient temperature by recording their Fourier transformed infrared spectra (FT-IR) and quantifying their contents of selected secondary volatile oxidation products (SVOPs). Thus, this research advances our understanding on the relation between the lipid oxidation of encapsulated fish oil and the encapsulation technology used to produce such encapsulates. The latter will allow the better design of dried omega-3 delivery systems with enhanced oxidative stability.

## 2. Materials and Methods

### 2.1. Materials

The refined fish oil (Omega Oil 1812 TG Gold) was purchased from BASF Personal Care and Nutrition GmbH (Illertissen, Germany) and stored at −80 °C until use. The Tween-20 (T20) was obtained from Sigma-Aldrich (Darmstadt, Germany) and CITREM (GRINDSTED*^®^* CITREM LR 10 EXTRA MT) were provided by Danisco (Copenhagen, Denmark). The pullulan (P) was kindly donated by Hayashibara Co., Ltd. (Okayama, Japan). The glucose syrup (GS; DE38, C*Dry 1934) was supplied by Cargill Germany GmbH (Krefeld, Germany). The maltodextrin (MD; DE21) and whey protein concentrate (ca. 35 wt% protein content) were generously donated by Abbott Laboratories S.A. (Granada, Spain). The whey protein concentrate hydrolysate (WPCH) used as an emulsifier was produced in an automatic titrator (718 Stat Titrino; Metrohm AG, Herisau, Switzerland) using Alcalase 2.4 L to a degree of hydrolysis of 10% (DH10), as described by Rahmani-Manglano et al. [16]. Then, the hydrolysate (WPCH) was freeze dried and stored at 4 °C until further use. The rest of the reagents used for the analysis were of analytical grade.

### 2.2. Production of the Spray-Dried Capsules

Fish-oil-in-water emulsions were produced by dispersing the fish oil (5 wt%) in the aqueous phase containing the encapsulating agent (GS or MD) and the emulsifier (WPCH or T20). The total solid content of the emulsions was fixed to 34 wt%, leading to a final oil load of the microcapsules of ca. 13 wt%. Therefore, depending on the emulsifier used (WPCH = 6 wt% or T20 = 0.35 wt%), the concentrations of the encapsulating agent varied (28 wt% or 34 wt%, respectively). The concentration of T20 was optimized to achieve a similar oil droplet size distribution to that of the WPCH-based emulsions (data not shown). The emulsions prepared with WPCH as the emulsifier had a final protein content of 2 wt%, resulting in a protein/oil ratio (P/O ratio) of 0.4. The spray-dried emulsions were produced as follows. First, a coarse emulsion was prepared by adding the oil to the aqueous phase during the first minute of mixing at 15,000 rpm using an Ultraturrax T-25 homogenizer (IKA, Staufen, Germany). The total mixing time was 2 min. Then, the pre-emulsion was homogenized in a high-pressure homogenizer (PandaPLUS 2000; GEA Niro Soavi, Lübeck, Germany) at a pressure range of 450/75 bar and by applying 3 passes. Right after production, the emulsions were subjected to spray-drying in a laboratory-scale spray-drier (Büchi B-190; Büchi Labortechnik, Flawill, Switzerland) at 180 and 90 °C for the inlet and outlet temperatures, respectively. The drying air flow was fixed to 25 Nm^3^/h.

### 2.3. Production of the Electrosprayed Capsules

#### 2.3.1. Monoaxially Electrosprayed Capsules

First, fish-oil-in-water emulsions were produced. The aqueous phase consisted of WPCH (4.3 wt%), P (3.0 wt%), and the main encapsulating agent (GS or MD, 15 wt%), which were dissolved in distilled water and stirred overnight (500 rpm) at ambient temperature. Then, the CITREM (1 wt%)/fish oil (3.7 wt%) mixture was dispersed in the aqueous phase using a rotor-stator homogenizer prior to high-pressure homogenization, as described in Section 2.2. The resulting fish-oil-in-water emulsions also had a P/O ratio of 0.4, and the final oil load of the microcapsules was ca. 13 wt%. Immediately after production, the emulsions were subjected to electrospraying. The electrospraying process was carried out using a system consisting of a drying chamber equipped with a variable high-voltage power supply (up to 30 kV), a syringe pump, and a 15 × 15 cm collector plate made of stainless steel (SpinBox Electrospinning; Bioinicia, Valencia, Spain). The emulsions were loaded into a 5 mL syringe, which was placed in the syringe pump and connected to the monoaxial emitter via a PTFE tube. A 16G needle (Proto Advantage, Hamilton, ON, Canada) was coupled to the monoaxial emitter and the needle tip was placed 15 cm apart from the collector plate (horizontal conformation). The flow rate (0.2 or 0.3 mL/h) and the voltage (17–20 kV) were optimized for each emulsion in order to avoid dripping and droplets in the collector. The electrospraying process was carried out at ambient temperature and ambient relative humidity (21–29 °C, 22–48% RH) in batches of 30 min. Between batches, the remaining emulsion was gently stirred (100 rpm) to minimize the physical destabilization of the emulsion and the oil droplet size was monitored during the processing time (0 h, 24 h, and 36 h) via laser diffraction in a Mastersizer 2000 system, (Malvern Instruments, Ltd., Worcestershire, UK) as described elsewhere [16]. The powder collected from the different batches was gently mixed before sampling to ensure that the analyzed samples were homogeneous and representative of the obtained material.

#### 2.3.2. Coaxially Electrosprayed Capsules

For the coaxial electrospraying, the biopolymer solution flowing through the annular gap between the inner and outer needles was produced following two different approaches. In the first approach, the encapsulating agent (GS or MD, 15 wt%), P (1 or 2 wt% for MD and GS, respectively), and T20 (1 wt%) were dissolved in distilled water and stirred overnight (500 rpm) at ambient temperature before electrospraying. In the second approach, the encapsulating agent (GS or MD, 15 wt%), P (3 wt%), and T20 (1 wt%) were dissolved in distilled water and stirred overnight (500 rpm). Afterwards, the biopolymer solution was passed through a high-pressure homogenizer (450/75 bar, 3 passes) prior to electrospraying. 

The electrospraying process was carried out in the setup described in Section 2.3.1, but this time a coaxial emitter consisting of two concentric needles was used. Thus, two syringe pumps working simultaneously were used. The neat fish oil was infused through the inner needle (ID/OD = 0.6/0.9 mm), and through the annular gap between needles flowed the biopolymer solution (outer needle 16G). The outer flow rate (F1) was fixed to 0.36 or 0.60 mL/h and the inner flow rate (F2) was adjusted to achieve a final oil load in the microcapsules of ca. 13 wt% (F2 = 0.012 or 0.021 mL/h, respectively). The voltages varied from 10 to 13.5 kV and from 16 to 18 kV depending on the flow rates used for the GS- or MD-based shell solutions. The flow rates and voltage combinations were optimized in order to avoid dripping and droplets in the collector. The process was conducted at ambient temperature and ambient relative humidity (21–30 °C, 23–51% RH) in batches of 60 min. The powders collected from the different batches were gently mixed before sampling to ensure that the analyzed samples were homogeneous and representative of the obtained material.

### 2.4. Characterization of the Capsules

#### 2.4.1. Morphology and Particle Size Distribution

The morphology of the capsules was investigated via scanning electron microscopy (SEM) using an FESEM microscope (LEO 1500 GEMINI, Zeiss, Germany). Depending on the microencapsulation technology used, a thin layer of microcapsules (i.e., spray-drying) or a piece of approximately 0.5 × 0.5 cm aluminum foil containing the sample (i.e., electrospraying) was placed on the carbon tape and carbon-coated using an EMITECH K975X Turbo-Pumped Thermal Evaporator (Quorum Technologies, UK). The SEM images were acquired in the range of 500×–15K× magnification with a 5 kV accelerating voltage. The particle size distributions and mean diameters were determined by measuring 180 randomly selected capsules using ImageJ software (National Institute of Health).

#### 2.4.2. Encapsulation Efficiency 

The encapsulation efficiency (EE) of the capsules was measured as described by Prieto and Lagaron [12] with some modifications. Approximately 25 mg of microcapsules was immersed in 10 mL of hexane and gently shaken for 30 s. Then, the mixture was filtered into a pyrex tube and the absorbance of the filtrate was measured at 250 nm in a UV-Vis double beam spectrophotometer (Thermo Spectronic Helios Alpha 9423 UVA 1002E, Thermo Fisher Scientific, Waltham, MA, USA). The amount of oil contained in the filtrate was determined from a calibration curve (R^2^ = 0.99) prepared by dissolving various quantities of fish oil in hexane (0.1–2.0 mg/mL). The EE and was calculated as follows:

(1) $$EE,\%=\frac{A-B}{A}\times 100$$

where A refers to the total oil load of the microcapsules (g) and B to the easily extractable oil (g). The measurements were carried out in triplicate. The total oil load of the microcapsules was determined by extracting the fish oil using a hexane/2-propanol (1:1, *v/v*) solvent. For the extraction, ca. 50 mg of powder was dissolved by adding 10 mL of distilled water, and the total oil load was determined by measuring the absorbance of the lipid extract, as previously described by Rahmani-Manglano et al. [17]. The measurements were carried out in triplicate.

### 2.5. Oxidative Stability 

To monitor the oxidative stability, 10 mg of capsules was stored in a 2 mL plastic Eppendorf tube at 25 °C in the dark for 6 weeks for the FT-IR analysis. For the measurement of secondary volatile oxidation products, 150 mg of capsules was stored in a 2 mL plastic Eppendorf tube at 25 °C in the dark for 6 weeks. Samples were taken at week 0, 2, 4, and 6 for the analysis.

#### 2.5.1. Fourier Transform Infrared Spectra (FT-IR) Analysis 

FT-IR spectra of the capsules were recorded on a JASCO FT/IR 6200 (Madrid, Spain) spectrometer operating in transmission mode. Approximately 1.5 mg of capsules was dispersed in ca. 150 mg of spectroscopic-grade potassium bromide (KBr) and subsequently ground. Then, a pellet was formed by compressing the mixture at ca. 150 MPa. All spectra were recorded within the wavenumber range of 4000–400 cm^−1^ by averaging 100 scans at 2 cm^−1^ resolution. The pellets were produced in triplicate for each sampling point and the measurements were carried out immediately after pellet production. For the raw ingredients used to produce the capsules (i.e., fish oil, GS, and T20), the attenuated total reflection (ATR)–FTIR spectra were recorded with the same equipment and conditions.

#### 2.5.2. Secondary Volatile Oxidation Products–Dynamic Headspace GC-MS

Approximately 50 mg of microcapsules and 5 mg of internal standard (4-methyl-1-pentanol, 30 µg/g water) were weighed out in a 100 mL pear-shaped bottle, to which 5 mL of distilled water and 1 mL of antifoam (Synperonic 800 µL/L water) were added. The bottle was heated to 45 °C in a water bath while being purged with nitrogen (flow 250 mL/min, 30 min). Volatile secondary oxidation products were trapped on Tenax GR tubes. The volatiles were desorbed again via heating (200 °C) in an Automatic Thermal Desorber (ATD-400, Perkin Elmer, Norwalk, CN, USA), cryofocused on a cold trap (−30 °C), and released again (220 °C), which led to a gas chromatograph (HP 5890IIA, Hewlett Packard, Palo Alto, CA, USA; Column: DB-1701, 30 m × 0.25 mm × 1.0 µm; J&W Scientific, Santa Clara, CA, USA). The oven program had an initial temperature of 45 °C for 5 min, increasing by 1.5 °C/min until 55 °C, 2.5 °C/min until 90 °C, and 12.0 °C/min until 220 °C, where the temperature was kept for 4 min. The individual compounds were analyzed using mass-spectrometry (HP 5972 mass-selective detector, Agilent Technologies, USA; electron ionization mode, 70 eV; mass-to-charge scan ratios between 30 and 250). The individual compounds were identified via both 186 MS library searches (Wiley 138 K, John Wiley and Sons, Hewlett-Packard) and using authentic external standards, which was quantified through calibration curves. The external standards employed were 2-ethylfuran, 1-penten-3-ol, hexanal, heptanal, and (E,E)-2,4-heptadienal (Sigma-Aldrich, Brøndby, Denmark), and the standard solutions were directly injected into the Tenax tubes. The samples were analyzed in triplicate.

### 2.6. Statistical Analysis 

The data were subjected to a one-way analysis of variance (ANOVA) using Statgraphics version 5.1 (Statistical Graphics Corp., Rockville, MD, USA). Tukey’s HSD multiple range test was used at the 95% confidence level (*p* < 0.05) to determine significant differences between mean values.

## 3. Results and Discussion 

### 3.1. Characterization of the Capsules

#### 3.1.1. Morphology and Particle Size Distribution 

**Figure 1:**
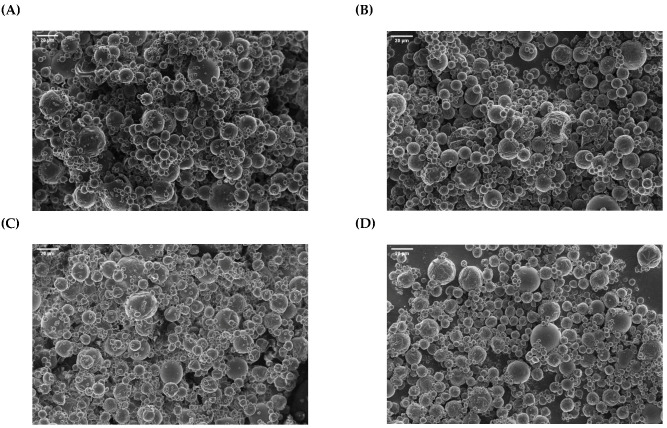
SEM images (**A**–**D**) of the spray-dried capsules loaded with fish oil: W-GS (**A**); T-GS (**B**); W-MD (**C**); T-MD (**D**). Scale bar: 20 µm.

**Figure 2:**
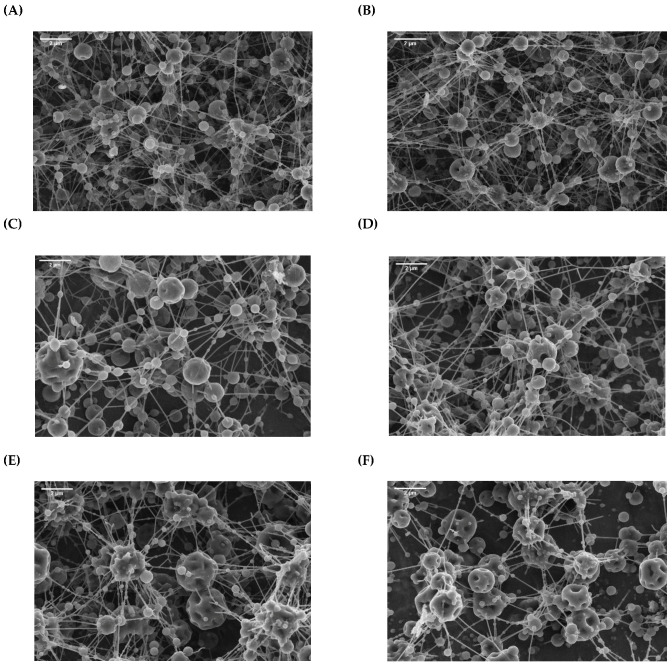
SEM images (**A**–**F**) of the electrosprayed capsules (monoaxial and coaxial) loaded with fish oil: GS-mo (monoaxial) (**A**); MD-mo (monoaxial) (**B**); GS-co (coaxial) (**C**); MD-co (coaxial) (**D**); GS-HPH-co (coaxial) (**E**); MD-HPH-co (coaxial) (**F**). Scale bar: 2 µm.

Figure 1 shows that spherical, non-agglomerated capsules were produced by spray-drying. Contrary to the discrete particles obtained for the spray-dried systems (Figure 1), thin fibers interconnecting the capsules could be observed in all the electrosprayed samples, irrespective of the emitter configuration (monoaxial or coaxial) (Figure 2). This has been attributed to the presence of pullulan in the different formulations, which is used as a “spin aid (bio)polymer” to increase the stability of the electrospraying process, allowing work at high flow rates (e.g., leading to increased throughput) [8]. Pullulan is an edible, water-soluble, non-ionic polysaccharide with great spinnability in water-based solutions [18], which has been previously used in the production of fish-oil-loaded nanofibers as the main biopolymer [10,19] or in the production of fish-oil-loaded nanocapsules within carbohydrate-based matrices as a thickening agent [11]. In the current study, the pullulan concentration was optimized in order to increase the viscoelasticity of the emulsions or solutions, allowing stable processing conditions at high flow rates (e.g., no dripping and droplets in the collector), but avoiding the formation of thick or strong fibers. Thus, the samples gathered from the collector plate had the appearance of a flowing powder, which led us to conclude that the fibrils observed were easily disrupted when subjected to mechanical forces (e.g., detachment of samples from the collector with a spatula). It should be noted that powder and capsules are preferred to fibers for the enrichment of food matrices due to their better dispersibility [20].

**Figure 3:**
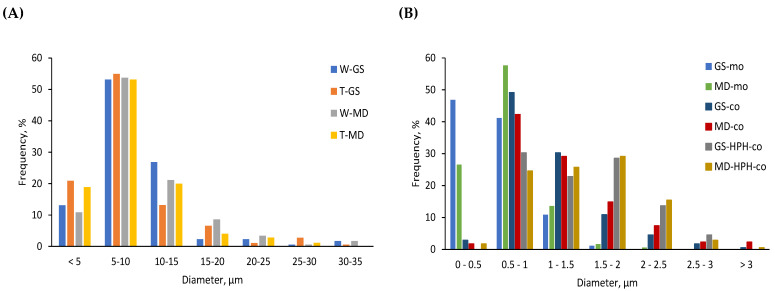
Particle size distribution of the capsules produced by spray-drying (**A**) and electrospraying (monoaxial and coaxial) (**B**).

Figure 3 shows the particle size distribution of the different capsules obtained in this study. No significant differences were observed in the mean diameter of the spray-dried capsules or in their particle size distribution regardless of the encapsulating agent (e.g., GS or MD) or the emulsifier (e.g., WPCH or T20) used in the formulation. The mean diameters of the spray-dried capsules obtained with GS or MD as encapsulating agents ranged from 8.57 ± 5.30 µm to 9.77 ± 5.36 µm (*p* > 0.05), and more than 90% of the particles had a size below 20 µm (Figure 3A). Moreover, it is noteworthy that the particle size distribution of the capsules produced by spray-drying was significantly wider (ranging from below 5 to over 35 µm) (Figure 3A) compared to that of the electrosprayed capsules (ranging from below 0.5 to over 3 µm) (Figure 3B). 

As in the case of spray-drying, the monoaxially electrosprayed capsules (GS-mo and MD-mo samples) (Figure 2A,B) did not show significant differences regarding their morphology and size, irrespective of the encapsulating agent employed (mean diameters of 0.6 ± 0.3 µm and 0.7 ± 0.3 µm for GS-mo and MD-mo, respectively). Furthermore, in line with the spray-dried capsules, the monoaxially electrosprayed capsules mostly showed spherical shapes with smooth surfaces, although some dented particles could be also spotted (Figure 2A,B). It is worth noting that more than 80% of the monoaxially electrosprayed capsules were below 1 µm (Figure 3B). These results are in line with those previously reported by García-Moreno et al. [11], who also produced spherical capsules with smooth surfaces when electrospraying fish-oil-in-water emulsions containing GS or dextran as encapsulating agents (70% of the capsules below 1 µm). 

Interestingly, despite the differences in the formulation of the biopolymer-based shell solutions (pullulan content) and the production process (with or without high-pressure homogenization, HPH), the morphologies of the coaxially electrosprayed capsules were fairly similar among the samples (Figure 2C–F). For the GS-co and MD-co samples (without HPH), the differences in the formulation of the shell solutions depended on the different molecular weights of the carbohydrates as a consequence of their dextrose equivalence (DE) values (DE38 for GS and DE21 for MD). Increasing the DE of a carbohydrate leads to smaller oligosaccharides with a lower molecular weight, which results in solutions with lower viscoelasticity at the same (bio)polymer concentration [21]. Hence, it was necessary to increase the pullulan concentration for the GS-based shell solution when compared to the MD-based solution in order to achieve a stable electrospraying process (2 wt% P for the GS-based shell solution over 1 wt% P for the MD-based shell solution). On the other hand, when the shell solution was subjected to HPH prior to electrospraying, the same content of pullulan in the GS- or MD-based formulations (3 wt% P) allowed the stabilization of the electrospraying process. It is noteworthy that despite the higher content of pullulan in the -HPH-co shell solutions, no differences were observed in the amount or thickness of the fibril defects between the coaxially electrosprayed capsules (Figure 2C–F), which is explained by the break of the pullulan polymer chains during homogenization at high pressures. 

Overall, regarding the electrosprayed systems (monoaxial and coaxial), it should be noted that coaxially electrosprayed capsules showed more and larger dents on the capsules’ surface compared to monoaxially electrosprayed systems (Figure 2). The morphology development of dried particles is highly influenced by the rheological properties of the skin, as well as the subsequent crust, formed during the drying process. Elastic skins are able to withstand internal and surface stresses that occur during drying, leading to smooth particles, whilst viscous skins deform, leading to wrinkled, folded, or dented particles [21]. LMW carbohydrates-based solutions have been reported to exhibit a viscous behavior upon drying, causing the deformation of the particle surfaces [21], thereby explaining the large dents observed in the coaxially electrosprayed systems compared to the monoaxially electrosprayed capsules. This is explained on the basis that for the monoaxial electrospraying, a fish-oil-in-water emulsion was infused to produce the capsules, whilst for the coaxial electrospraying, the shell processing solution consisted of a GS- or MD-based solution. Moreover, significantly larger capsules were produced via coaxial electrospraying over monoaxial electrospraying (Figure 3B). This was related to the electrospraying processing conditions, especially the operating flow rates. At high infusing flow rates, larger droplets are formed at the tip of the emitter, meaning larger capsules are obtained after drying. Hence, the -mo capsules (ca. 85% of the capsules < 1 µm) were smaller than the -co capsules (ca. 45% of the capsules < 1 µm), which at the same time were smaller than the -HPH-co capsules (ca. 25% of the capsules < 1 µm). Stable processing conditions could be achieved at higher flow rates for the -HPH-co systems (over the -co systems) due to their higher pullulan content in the shell solution formulation (0.36 mL/h for -co systems and 0.60 mL/h for -HPH-co systems). The latter, together with the differences in the solid contents of the shell solutions (i.e., 16 wt% solids for MD-co over 18 wt% solids for MD-HPH-co), explains the differences observed in the particle size (*p* < 0.05) and throughput of the process (from ca. 80 mg/h for -co capsules to ca. 143 mg/h for -HPH-co capsules). Nonetheless, although the infusion flow rate increased by up to two times for the production of -HPH-co systems compared to -co systems, the size of the capsules did not increase in the same proportion as a consequence of the higher voltage applied during the -HPH-co capsule production process (ca. 18 kV for -HPH-co over ca. 13.5 kV for -co electrospraying). Higher processing voltages favor jet breakage into smaller droplets due to increased electrostatic charge repulsion, leading to smaller capsules after solvent evaporation [8].

#### 3.1.2. Encapsulation Efficiency (EE) 

**Figure 4:**
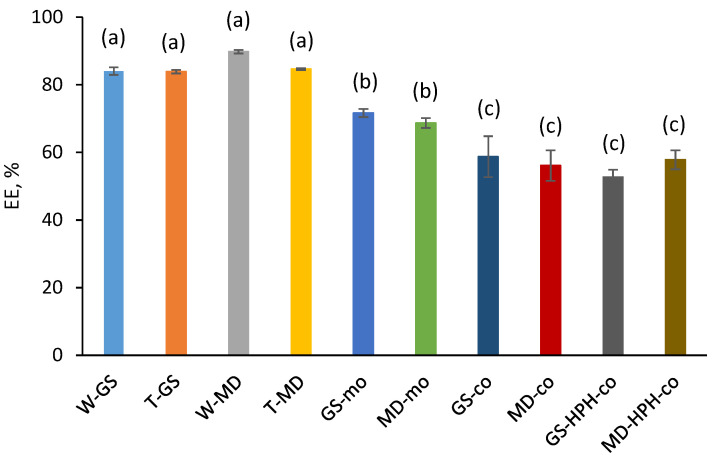
Encapsulation efficiency (EE) levels of the fish-oil-loaded capsules produced via spray-drying or electrospraying (monoaxial and coaxial). Samples followed by a letter (a–c) indicate statistical differences (*p* ≤ 0.05) between microcapsules.

Significant differences (*p* < 0.05) were observed in the EE values depending on the encapsulation technique used to produce the fish-oil-loaded capsules (i.e., spray-drying, monoaxial electrospraying, or coaxial electrospraying) (Figure 4). The highest EE was obtained for the spray-dried systems (EE = 84–90%), followed by the monoaxially electrosprayed (EE = 69–72%) and coaxially electrosprayed systems (EE = 53–59%), respectively. 

For emulsion-based encapsulation techniques, such as spray-drying and monoaxial electrospraying, the EE is closely related to the infeed emulsion formulation and its physical stability. The drying of physically stable monodisperse emulsions of small oil droplets results in the better entrapment of the oil within the encapsulating matrix [22,23]. Furthermore, it has been reported that for a fixed oil load, increasing the wall material concentration improves the EE [6]. Therefore, the differences in the EE values reported for the spray-drying and monoaxial electrospraying encapsulation techniques could be related to the different emulsion formulations and their characteristics before and during processing (e.g., oil droplet size). From our previous work, it could be observed that all emulsions fed to the spray-drier presented a monomodal oil droplet size distribution, with the D[4,3] values ranging from 0.45 ± 0.01 µm to 0.56 ± 0.01 µm [17]. Conversely, the emulsions subjected to monoaxial electrospraying in this study showed both wider (GS-mo) and bimodal (MD-mo) droplet size distributions before processing (see Figure S1 in the Supplementary Materials), as well as higher D[3,4] values, meaning larger oil droplets (D[3,4] = 1.01 ± 0.34 µm and 1.60 ± 0.54 µm, respectively).

For monoaxial electrospraying, the emulsions were produced by dispersing the oil phase, consisting of a CITREM (1 wt%)/fish oil (3.7 wt%) mixture, within the aqueous phase, containing the encapsulating agent (GS or MD, 15 wt%), the emulsifier (WPCH, 4.3 wt%), and pullulan (3 wt%). Both the presence of pullulan in the aqueous phase and CITREM in the oil phase may have increased their respective viscosities to an extent, resulting in poorer emulsification, leading to emulsions of larger oil droplets. Moreover, although the electrosprayed emulsions were gently stirred during the total processing time to minimize their physical destabilization, this was not totally achieved (see Figure S1 in the Supplementary Materials). After 36 h, the GS-mo emulsion showed a relatively wider monomodal oil droplet size distribution and the main peak was displaced to larger diameter values when compared to at 0 h (main peak centered at ca. 3 µm after 36 h) (see Figure S1A of the Supplementary Materials). Likewise, for the MD-mo emulsion, the proportion of the first peak of the bimodal droplet size distribution decreased as the proportion of the second peak increased and was progressively displaced to larger diameter values (second peak centered at ca. 5 µm after 36 h; see Figure S1B of the Supplementary Materials). These results suggest that the electrosprayed emulsions suffered physical destabilization during processing, favoring the presence of easily extractable surface oil in the electrosprayed capsules. In addition, for a fixed oil load, an increased particle size implies thicker encapsulating walls, resulting in better entrapment of the oil droplets within the encapsulating matrix [23]. Therefore, the significantly different capsules sizes (*p* < 0.05) as a result of the different atomization processes of both techniques could further explain the lower EE values reported for the -mo systems over the spray-dried capsules (*p* < 0.05).

Nonetheless, the EE values reported for the capsules produced by monoaxial electrospraying in the current work contrast with previous studies on the encapsulation of fish oil by monoaxial electrospraying within carbohydrate-based matrices. García-Moreno et al. [10,11] produced fish-oil-loaded microcapsules using dextran or GS as the encapsulating agents, with EE values ranging from 76 to 92%, depending on the production process (conventional electrospraying or electrospraying assisted by pressurized gas, EAPG). Likewise, Prieto and Lagaron [12] reported similar EE values when encapsulating algae oil within a maltodextrin-based matrix using EAPG (EE = 77% for an oil load of 10 wt%). It should be noted, however, that finer emulsions were produced in the aforementioned studies [10,11,12].

The coaxially electrosprayed capsules presented the lowest EE values (EE = 53–59%), regardless of the shell solution formulation or production process (-co or -HPH-co capsules) (*p* > 0.05) (Figure 4). These low EE values could be attributed to the lack of surface-active properties of the LMW carbohydrates used as encapsulating agents (i.e., GS or MD) [24], which could have resulted in reduced entrapment of the fish oil within the encapsulating matrix during processing. Moreover, the lower EE values are in agreement with the large dents observed on the surfaces of the coaxially electrosprayed capsules (Figure 2C–F), which were related to the presence of non-encapsulated oil droplets and to higher access of the extracting solvent to the encapsulated oil once the non-encapsulated oil fraction is removed [25]. These results highlight that the outcome of the coaxial electrospraying process is influenced by the properties of the encapsulating agent/s used and that ingredients with amphiphilic properties are required to improve the retention properties of the encapsulating wall, especially when neat fish oil is infused as the core solution.

Overall, our EE results for the electrospraying processes (monoaxial vs. coaxial) contrast with those reported by other authors [14,26]. Pérez-Masiá et al. [26] found that the coaxially electrosprayed capsules resulted in higher EE (based on intact lycopene) compared to those produced by monoaxial electrospraying when dextran was used as the encapsulating agent (EEcoaxial = 58% over EEmonoaxial = 26%). The authors attributed this to the low physical stability of the emulsion during monoaxial processing. Nonetheless, it should be noted that the EE value reported by the aforementioned authors for the coaxially electrosprayed system using carbohydrates as wall materials to encapsulate a hydrophobic bioactive is in line with the EE values obtained in the current work (EEcoaxial = 53–59%). Similarly, Gómez-Mascaraque et al. [14] produced α-linolenic acid (ALA)-loaded microcapsules within protein-based matrices (i.e., zein) via monoaxial and coaxial electrospraying. These authors found higher EE values for the coaxial encapsulates (EEcoaxial = ca. 90% over EEmonoaxial = ca. 70%) when using an extra shell layer based on zein, but no significant differences were observed when gelatin was employed as the extra shell layer. Furthermore, contrary to our study, the core material that these authors used was a zein/ALA solution and not only ALA. This could explain the higher EE values reported for the zein-based coaxially electrosprayed systems, since the resulting capsules consisted of an outer zein-based layer coating the inner zein-containing matrix, leading to better bioactive entrapment due to the surface-active and encapsulating properties of zein [27]. Interestingly, the EE value reported for the monoaxial zein/ALA system by Gómez-Mascaraque et al. [14] is in line with the EE values reported in this study when fish-oil-in-water emulsions stabilized with WPCH were electrosprayed in the monoaxial configuration (EEmonoaxial = 69–72%). WPCH has been proven to possess great emulsifying and film-forming properties [15,16], which may have favored the monoaxial electrospraying outcome by (i) stabilizing the fish oil droplets in the emulsion during processing and (ii) increasing the biopolymer-based wall matrix retention properties during and after drying.

### 3.2. Oxidative Stability of the Capsules

#### 3.2.1. FT-IR

First, the oxidative stability of the different types of capsules was evaluated via FT-IR by monitoring the changes in the characteristic absorption bands of omega-3 PUFAs during the storage time. Although information on the initial oxidation status of the encapsulated oil is not provided by FT-IR analysis, this technique has been proven to be a useful tool to evaluate the oxidation stage of neat edible oils [28,29] and has been extensively used to monitor the oxidative stability of capsules loaded with omega-3 PUFA-rich oils produced either by spray-drying [30,31] or electrospraying (monoaxial and coaxial) [11,12,14].

For omega-3 PUFA-rich oils, the characteristic absorption band corresponds to the stretching of cis-alkene groups (–HC=CH–) at 3012 cm^−1^, and a decrease in the intensity of this band (due to the loss of cis double bonds) has been proposed as a marker of lipid oxidation [28]. Thus, the intensity of this band was monitored during the storage time. The band at 1456 cm^−1^, assigned to rocking vibrations of C–H bonds of cis-disubstituted alkenes, was used as the internal standard for normalization purposes, since this band remains unchanged during the lipid oxidation of omega-3 PUFA-rich oils (e.g., algae oil rich in DHA), as reported by other authors [12]. Therefore, for each sampling point, the relative absorbance was calculated (A_3012_/A_1456_) and the results were normalized to the initial relative absorbance value (week 0) for a better comparison among the samples. The ATR-FTIR spectra of the fish oil, the GS, and the T20 used in the study are shown in Figure S2 in the Supplementary Materials, where it can be seen that the band at 3012 cm^−1^ does not overlap with the infrared bands of the other ingredients. The spectra of the rest of the ingredients used in the production of the capsules also did not overlap with the fish oil band at 3012 cm^−1^, as can be seen elsewhere [10,12].

**Figure 5:**
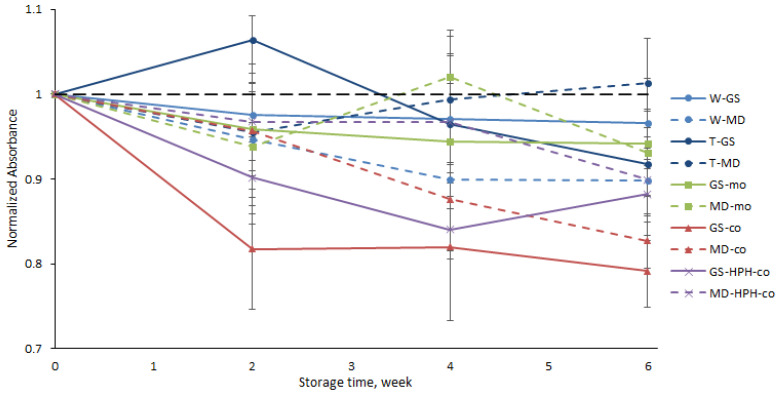
Oxidative stability levels measured using FT-IR of the of the fish-oil-loaded capsules produced by spray-drying or electrospraying (monoaxial and coaxial) during storage.

Overall, a slight decrease in the normalized absorbance values was observed during storage for all samples (Figure 5), which indicates that the capsules produced by the different encapsulation techniques were not extensively oxidized based on the reduction in the characteristic omega-3 absorption band (at 3012 cm^−1^). Likewise, García-Moreno et al. [11] did not observe a significant decrease in the normalized absorbance of the band at 3012 cm^−1^ in fish-oil-loaded capsules produced via monoaxial electrospraying using dextran or GS as the encapsulating biopolymer after 21 days of storage at 20 °C. Nonetheless, Figure 5 shows a more pronounced decrease in the normalized absorbance for GS-co and MD-co samples, indicating that these capsules were the less oxidatively stable during storage, while no clear differences could be observed for the rest of the systems, regardless of the encapsulating agent used or the encapsulation technique (i.e., spray-dried, -mo, and -HPH-co systems). Low oxidative stability in encapsulated oils is often related to low EE values, since unprotected surface oil is extremely prone to oxidation due to direct contact with prooxidant species (e.g., oxygen) [25]. However, the lower oxidative stability observed in the -co systems cannot be only attributed to a higher content of easily oxidized surface oil, since the EE values reported for the -HPH-co capsules were not significantly different (*p* > 0.05) (Figure 4). Therefore, our results show that although the HPH treatment of the shell solution prior to coaxial electrospraying did not improve the oil retention properties of the encapsulating wall during processing, it did improve the oxidative stability of the -HPH-co systems over the -co systems. The latter suggests that the physicochemical properties of the coaxial capsules (e.g., particle size, thickness of the encapsulating wall, permeability to oxygen) significantly influenced the lipid oxidation rather than the non-encapsulated oil, which will be further discussed below.

#### 3.2.2. Secondary Volatile Oxidation Products (SVOPs)

**Figure 6:**
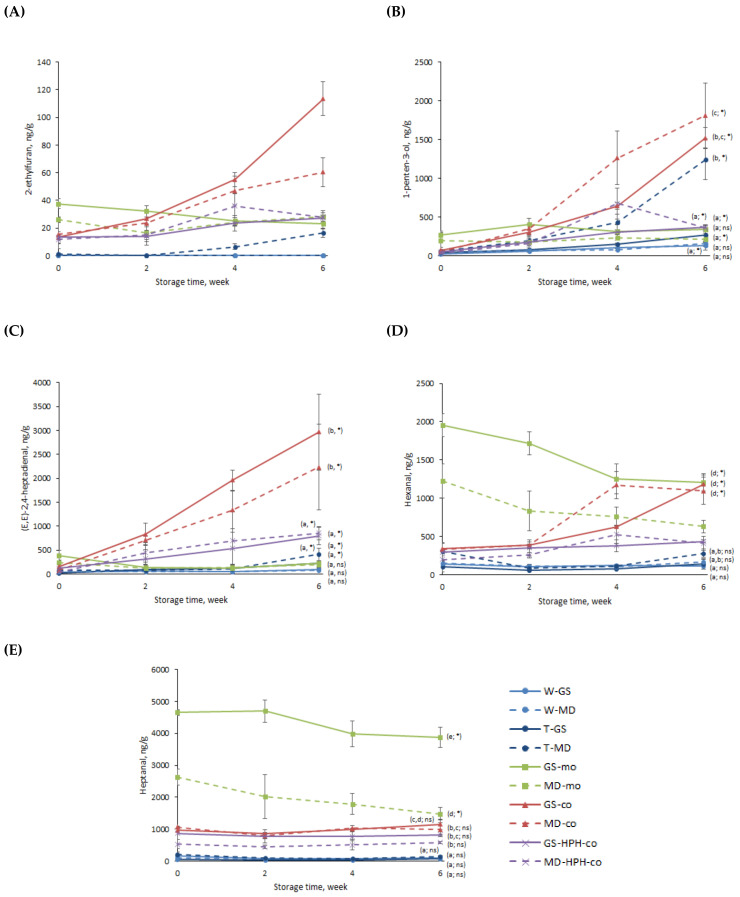
Secondary volatile oxidation products (SVOPs) of the fish-oil-loaded capsules produced by spray-drying or electrospraying (monoaxial and coaxial) during storage: 2-ethylfuran (**A**); 1-penten-3-ol (**B**); (E,E)-2,4-heptadienal (**C**); hexanal (**D**); heptanal (**E**). Samples followed by a letter (a–e) indicate statistical differences (*p* ≤ 0.05) between capsules. Means within the same sample followed by an asterisk (*) indicate statistical differences (*p* ≤ 0.05) between week 0 and week 6. Means within the same sample followed by “ns” indicate no statistical differences (*p* > 0.05) between week 0 and week 6.

To further investigate the oxidative stability of the fish-oil-loaded capsules, the contents of selected secondary volatile oxidation products (SVOPs) were measured during 6 weeks of storage at 25 °C. (Figure 6). Volatiles such as 2-ethylfuran, 1-penten-3-ol, and (E,E)-2,4-heptadienal are typical compounds derived from the oxidation of omega-3 PUFAs related to unpleasant odors (e.g., flower, sweet or green) at low threshold values (e.g., 1-penten-3-ol = 0.5–3 ppm) [3]. Hexanal and heptanal, despite being compounds derived from the oxidation of omega-6 and omega-9 fatty acids, respectively, have also been identified in oxidized fish oils [11,32]. The odors of these SVOPs are described as grassy, fruity, green, or sharp, and their threshold values are also considerably low (0.014–1 ppm) [32].

Considering the results obtained for the SVOPs derived from the oxidation of omega-3 PUFAs (Figure 6A–C), the same trend as for the FT-IR analysis can be observed—the most oxidized samples after storage were the -co systems, whilst for the rest of the capsules (i.e., -HPH-co, -mo, and spray-dried capsules) no significant differences could be observed at the end of the storage time (Figure 6A–C; *p* > 0.05). Furthermore, irrespective of the SVOP considered (i.e., 2-ethylfuran, 1-penten-3-ol, and (E,E)-2,4-heptadienal), the same oxidation trend could be observed for the -co capsules. First, a slight increase in the concentration occurred during the first two weeks of storage, followed by a sharp increase from week 2 onwards (Figure 6A–C). Nonetheless, as previously discussed, the lower oxidative stability found for the -co capsules cannot only be attributed to their high content of surface oil, since the EE values reported for the coaxially electrosprayed systems (i.e., -co and -HPH-co systems) were not significantly different among the samples (Figure 4; *p* > 0.05).

Oxygen’s solubility and diffusion through the encapsulating wall have been proven to play a key role in the lipid oxidation of encapsulated fish oil [33]. Both are influenced by several factors, such as the type of encapsulating (bio)polymer and its properties (e.g., molecular weight and permeability to oxygen), the thickness of the encapsulating wall (e.g., solids content, oil load), and the capsule size (e.g., surface-to-volume ratio) [33]. Therefore, the different oxidation rates and extents observed in the coaxially electrosprayed systems (i.e., -co and -HPH-co systems) could be attributed to the different physicochemical properties of the capsules influencing the oxygen diffusivity. Smaller capsules, as is the case for -co systems compared to -HPH-co capsules (Figure 3B; *p* < 0.05), result in an increased contact area with environmental prooxidants (e.g., oxygen), which facilitates their diffusion through the encapsulating matrix, thereby promoting lipid oxidation [33]. Additionally, for a fixed oil load, smaller capsules result in thinner and, therefore, more permeable encapsulating walls. This, together with the higher content of pullulan in the -HPH-co capsules, may explain their higher oxidative stability, since pullulan acts as an efficient oxygen barrier due to its intrinsic impermeability to oxygen [34]. Moreover, the breakage of the pullulan chains during HPH for -HPH-co samples may have contributed to the formation of a more densely packed dry matrix, which may have limited the oxygen diffusion to a higher extent, although the latter requires further research. Nonetheless, the significantly higher oxidation rate and extent observed in -co systems cannot only be attributed to the physicochemical properties of the capsules in regard to their size and the thickness of the encapsulating wall. Contrary to emulsion-based methods, which lead to capsules consisting of small discrete oil droplets distributed within the dried encapsulating matrix, coaxially electrosprayed capsules consist, theoretically, of an oil droplet located in the core coated with the dried encapsulating wall. It is well known that lipid oxidation occurs as a chain reaction between lipid radicals and oxygen until it is interrupted either by the action of an external agent (e.g., antioxidant) or by the unavailability of an oxygen or hydrogen source [3]. Therefore, whilst emulsion-based encapsulates will oxidize progressively as the oxygen diffuses from the surface to the core, reaching the different oil droplets, lipid oxidation will propagate faster and more easily in coaxially electrosprayed systems once the oxygen reaches the oily core. This could be a further indication that for -HPH-co capsules, the oxygen diffusivity through the encapsulating matrix was prevented more efficiently than for -co capsules, since although lipid oxidation occurred, these samples were significantly less degraded at the end of the storage time, showing a lipid oxidation extent comparable to that of the emulsion-based encapsulates (Figure 6A–C).

Interestingly, the monoaxially electrosprayed capsules were the most oxidized after processing, as shown by their significantly higher content in the selected volatiles studied at the beginning of the storage time (Figure 6 and see also Figure S3A in the Supplementary Materials; *p* < 0.05). As previously mentioned, the monoaxially electrosprayed emulsions contained CITREM, which according to the supplier, was made from refined sunflower oil. Sunflower oil is rich in omega-6 PUFAs, and both hexanal and heptanal have been identified in neat sunflower oil [35]. In addition, the sunflower oil emulsification process favors both the generation and release of SVOPs [36], which could explain the significantly higher initial concentrations of hexanal and heptanal found in the -mo capsules after production (Figure 6D,E, *p* < 0.05). In addition, the monoaxially electrosprayed emulsions were stored between batches for a total processing time of 36 h, which resulted in physical destabilization, and consequently in a relatively high content of non-encapsulated surface oil (Figure 4). Indeed, it has been reported that the oxidative stability of emulsions is directly related to their physical stability, with fine and physically stable emulsions being less prone to lipid oxidation [15]. Therefore, the higher content of SVOPs found in the -mo capsules at the beginning of the storage time (Figure 6) could be attributed to the production and subsequent storage of the emulsions during processing and to the lipid oxidation of the non-encapsulated oil fraction during the processing time between batches. Conversely, spray-dried emulsions were dried right after production, and the spray-drying process led to capsules with better retention properties, as confirmed by their higher EE values (Figure 4). This, together with the short residence time of the spray-dried capsules in the drying chamber, could explain their low contents in the selected volatiles at the beginning of the storage time (see Figure S3 in the Supplementary Materials). Likewise, low initial lipid oxidation was observed for -HPH-co systems (see Figure S3 in the Supplementary Materials), which has been attributed to the absence of the emulsification step in the production of these systems. Nonetheless, despite the higher content of the selected volatiles found at the beginning of the storage time for -mo systems, their concentration did not significantly increase during storage (see Figure S3 in the Supplementary Materials). The monoaxially electrosprayed capsules (i.e., GS-mo and MD-mo) contained WPCH and pullulan in the formulation. WPCH possesses both film-forming and antioxidant properties (e.g., radical scavenging and metal chelating), and combined with LMW carbohydrates as bulk materials, this leads to highly stable capsules in terms of lipid oxidation, as confirmed in our previous work [16]. This, together with the intrinsic oxygen-impermeability of pullulan and the breakage of the pullulan chains during the emulsification process, may have contributed to the formation of a highly dense encapsulating matrix with enhanced impermeable properties, which could explain why the initial contents of the volatiles did not increase during storage. It is noteworthy, however, that the concentrations of both hexanal and heptanal significantly decreased for the -mo systems during the course of the lipid oxidation (Figure 6D,E; *p* < 0.05). This could be explained on the basis of aldehyde decomposition as a result of the so called non-enzymatic browning reactions taking place in the presence of the WPCH amino groups [37], as previously observed by other authors [38,39].

Finally, high oxidative stability was observed for the spray-dried capsules, which showed both low initial and low final contents for all selected volatiles (Figure 6). As previously mentioned, the use of spray-drying technology resulted in large capsules (Figure 3A), leading to (i) thicker encapsulating walls for the same oil load and (ii) a significantly reduced surface-to-volume ratio, both affecting the oxygen diffusivity through the encapsulating wall. In addition, the high EE values reported for these samples confirmed the low content of easily oxidized non-encapsulated surface oil. Furthermore, substituting WPCH for T20 as the emulsifier did not significantly affect the overall oxidative stability of the spray-dried systems, except for the T-MD sample (see Figure S3 in the Supplementary Materials). The latter has been attributed to the higher molecular weight of the MD due to its lower DE, which may have resulted in a more porous encapsulating wall. Additionally, contrary to the WPCH, T20 lacks antioxidant properties, meaning lipid oxidation occurring at the oil–encapsulating agent interface could not be prevented.

## 4. Conclusions

To the best of the authors’ knowledge, neat fish-oil-loaded capsules were for the first time produced via coaxial electrospraying using LMW carbohydrates (i.e., glucose syrup (GS) or maltodextrin (MD)) as the encapsulating agents. Furthermore, in the study, fish-oil-loaded capsules were also produced by spray-drying and monoaxial electrospraying in the presence of GS or MD, using whey protein concentrate hydrolysate (WPCH) as the emulsifier. Our results show that the physicochemical properties of the capsules (e.g., the particle size or EE) were significantly influenced by the encapsulation technology. As expected, larger capsules were produced using spray-drying over electrospraying technology, and the particle size distribution was also broader. Moreover, the use of emulsion-based encapsulation methods (i.e., spray-drying and monoaxial electrospraying) resulted in better entrapment of the fish oil within the encapsulating matrix (EE > 69%) compared to coaxial electrospraying (EE = 53–59%), due to the lack of surface-active properties of the main encapsulating agents used (i.e., GS or MD). This explained the lowest oxidative stability observed for the coaxially electrosprayed capsules. However, despite the higher content of non-encapsulated oil in the aforementioned systems, their oxidative stability could be significantly enhanced by increasing the content of pullulan in the shell solution. This was attributed to the larger size of the resulting capsules and to the higher content of pullulan in the encapsulating matrix, both influencing the oxygen diffusivity through the encapsulating wall. The use of emulsion-based encapsulation methods led to capsules with high oxidative stability, as confirmed by the low contents of SVOPs (i.e., 2-ethylfuran, (E,E)-2,4-heptadienal) found at the end of the storage time. Nonetheless, the monoaxially electrosprayed capsules were the most oxidized right after production due to the emulsification process, together with a larger time span between the emulsion production and drying.

## Data Availability

Not applicable.

## Supplementary Materials

The following supporting information can be downloaded at: https://www.mdpi.com/article/10.3390/antiox12020266/s1. Figure S1: Oil droplet size distribution of the fish-oil-loaded emulsions produced for monoaxial electrospraying during processing (0 h, 24 h, 36 h): GS-mo (A) and MD-mo (B). Figure S2: ATR-FTIR spectra of the fish oil (solid line), glucose syrup (dashed line), and Tween-20 (dotted line). Figure S3: Secondary volatile oxidation products (SVOPs) of the fish-oil-loaded capsules produced by spray-drying or electrospraying (monoaxial and coaxial) at week 0 and week 6 of storage: 2-ethylfuran (A); 1-penten-3-ol (B); (E,E)-2,4-heptadienal (C); hexanal (D); heptanal (E).

## Abbreviations

PUFAs, polyunsaturated fatty acids; EPA, eicosapentaenoic acid; DHA, docosahexaenoic acid; ALA, α-linolenic acid; EE, encapsulation efficiency; LMW, low molecular weight; DE, dextrose equivalence; GS, glucose syrup; MD, maltodextrin; WPCH, whey protein concentrate hydrolysate; T20, Tween-20; P, pullulan; FT-IR, Fourier transformed infrared spectra; SVOPs, secondary volatile oxidation products; SEM, scanning electron microscopy; HPH, high-pressure homogenization.

## References

[1] Djuricic I., Calder P.C. (2021). Beneficial Outcomes of Omega-6 and Omega-3 Polyunsaturated Fatty Acids on Human Health: An Update for 2021. Nutrients.

[2] Ghelichi S., Hajfathalian M., García-Moreno P.J., Yesiltas B., Moltke-Sørensen A.-D., Jacobsen C., García-Moreno P.J., Jacobsen C., Sørensen A.-D.M., Yesiltas B. (2021). Food Enrichment with Omega-3 Polyunsaturated Fatty Acid. Omega-3 Delivery Systems.

[3] Rahmani-Manglano N.E., García-Moreno P.J., Espejo-Carpio F.J., Pérez-Gálvez A.R., Guadix-Escobar E.M., Aboudzadeh M.A. (2020). The Role of Antioxidants and Encapsulation Processes in Omega-3 Stabilization. Emulsion-Based Encapsulation of Antioxidants. Food Bioactive Ingredients..

[4] Turchiuli C., Jimenez Munguia M.T., Hernandez Sanchez M., Cortes Ferre H., Dumoulin E. (2014). Use of Different Supports for Oil Encapsulation in Powder by Spray Drying. Powder Technol..

[5] Acevedo-Fani A., Guo Q., Nasef N., Singh H., García-Moreno P.J., Jacobsen C., Sørensen A.-D.M., Yesiltas B. (2021). Aspects of Food Structure in Digestion and Bioavailability of LCn-3PUFA-Rich Lipids. Omega-3 Delivery Systems.

[6] Drosou C.G., Krokida M.K., Biliaderis C.G. (2017). Encapsulation of Bioactive Compounds through Electrospinning/Electrospraying and Spray Drying: A Comparative Assessment of Food-Related Applications. Dry. Technol..

[7] Serfert Y., Drusch S., Schwarz K. (2009). Chemical Stabilisation of Oils Rich in Long-Chain Polyunsaturated Fatty Acids during Homogenisation, Microencapsulation and Storage. Food Chem..

[8] García-Moreno P.J., Rahmani-Manglano N.E., Chronakis I.S., Guadix E.M., Yesiltas B., Sørensen A.-D.M., Jacobsen C., García-Moreno P.J., Jacobsen C., Sørensen A.-D.M., Yesiltas B. (2021). Omega-3 Nano-Microencapsulates Produced by Electrohydrodynamic Processing. Omega-3 Delivery Systems. Production, Physical Characterization and Oxidative Stability.

[9] Jaworek A., Sobczyk A.T. (2008). Electrospraying Route to Nanotechnology: An Overview. J. Electrostat..

[10] García-Moreno P.J., Özdemir N., Stephansen K., Mateiu R.V., Echegoyen Y., Lagaron J.M., Chronakis I.S., Jacobsen C. (2017). Development of Carbohydrate-Based Nano-Microstructures Loaded with Fish Oil by Using Electrohydrodynamic Processing. Food Hydrocoll..

[11] García-Moreno P.J., Pelayo A., Yu S., Busolo M., Lagaron J.M., Chronakis I.S., Jacobsen C. (2018). Physicochemical Characterization and Oxidative Stability of Fish-oil-loaded Electrosprayed Capsules: Combined Use of Whey Protein and Carbohydrates as Wall Materials. J. Food Eng..

[12] Prieto C., Lagaron J.M. (2020). Nanodroplets of Docosahexaenoic Acid-Enriched Algae Oil Encapsulated within Microparticles of Hydrocolloids by Emulsion Electrospraying Assisted by Pressurized Gas. Nanomaterials.

[13] Loscertales I.G., Barrero A., Guerrero I., Cortijo R., Marquez M., Gañán-Calvo A.M. (2002). Micro/Nano Encapsulation via Electrified Coaxial Liquid Jets. Science.

[14] Gómez-Mascaraque L.G., Tordera F., Fabra M.J., Martínez-Sanz M., Lopez-Rubio A. (2019). Coaxial Electrospraying of Biopolymers as a Strategy to Improve Protection of Bioactive Food Ingredients. Innov. Food Sci. Emerg. Technol..

[15] Padial-Domínguez M., Espejo-Carpio F.J., García-Moreno P.J., Jacobsen C., Guadix E.M. (2020). Protein Derived Emulsifiers with Antioxidant Activity for Stabilization of Omega-3 Emulsions. Food Chem..

[16] Rahmani-Manglano N.E., González-Sánchez I., García-Moreno P.J., Espejo-Carpio F.J., Jacobsen C., Guadix E.M. (2020). Development of Fish-oil-loaded Microcapsules Containing Whey Protein Hydrolysate as Film-Forming Material for Fortification of Low-Fat Mayonnaise. Foods.

[17] Rahmani-Manglano N.E., Tirado-Delgado M., García-Moreno P.J., Guadix A., Guadix E.M. (2022). Influence of Emulsifier Type and Encapsulating Agent on the in Vitro Digestion of Fish-oil-loaded Microcapsules Produced by Spray-Drying. Food Chem..

[18] Stijnman A.C., Bodnar I., Hans Tromp R. (2011). Electrospinning of Food-Grade Polysaccharides. Food Hydrocoll..

[19] García-Moreno P.J., Damberg C., Chronakis I.S., Jacobsen C. (2017). Oxidative Stability of Pullulan Electrospun Fibers Containing Fish Oil: Effect of Oil Content and Natural Antioxidants Addition. Eur. J. Lipid Sci. Technol..

[20] Jacobsen C., García-Moreno P.J., Mendes A.C., Mateiu R.V., Chronakis I.S. (2018). Use of Electrohydrodynamic Processing for Encapsulation of Sensitive Bioactive Compounds and Applications in Food. Annu. Rev. Food Sci. Technol..

[21] Siemons I., Politiek R.G.A., Boom R.M., van der Sman R.G.M., Schutyser M.A.I. (2020). Dextrose Equivalence of Maltodextrins Determines Particle Morphology Development during Single Sessile Droplet Drying. Food Res. Int..

[22] Paximada P., Howarth M., Dubey B. Electrosprayed Particles Derived from Nano-Emulsions as Carriers of Fish Oil. Proceedings of the TechConnect World Innovation Conference & Expo.

[23] Ramakrishnan S., Ferrando M., Aceña-Muñoz L., Mestres M., de Lamo-Castellví S., Güell C. (2014). Influence of Emulsification Technique and Wall Composition on Physicochemical Properties and Oxidative Stability of Fish Oil Microcapsules Produced by Spray Drying. Food Bioproc. Tech..

[24] Hogan S.A., McNamee B.F., O’Riordan E.D., O’Sullivan M. (2001). Emulsification and Microencapsulation Properties of Sodium Caseinate/Carbohydrate Blends. Int. Dairy, J..

[25] Drusch S., Berg S. (2008). Extractable Oil in Microcapsules Prepared by Spray-Drying: Localisation, Determination and Impact on Oxidative Stability. Food Chem..

[26] Pérez-Masiá R., Lagaron J.M., Lopez-Rubio A. (2015). Morphology and Stability of Edible Lycopene-Containing Micro- and Nanocapsules Produced Through Electrospraying and Spray Drying. Food Bioproc. Tech..

[27] Gómez-Mascaraque L.G., López-Rubio A. (2016). Protein-Based Emulsion Electrosprayed Micro- and Submicroparticles for the Encapsulation and Stabilization of Thermosensitive Hydrophobic Bioactives. J. Colloid Interface Sci..

[28] Guillén M.D., Cabo N. (1999). Usefulness of the Frequency Data of the Fourier Transform Infrared Spectra to Evaluate the Degree of Oxidation of Edible Oils. J. Agric. Food Chem..

[29] Guillen M.D., Cabo N. (2000). Some of the Most Significant Changes in the Fourier Transform Infrared Spectra of Edible Oils under Oxidative Conditions. J. Sci. Food Agric..

[30] Binsi P.K., Nayak N., Sarkar P.C., Jeyakumari A., Muhamed Ashraf P., Ninan G., Ravishankar C.N. (2017). Structural and Oxidative Stabilization of Spray Dried Fish Oil Microencapsulates with Gum Arabic and Sage Polyphenols: Characterization and Release Kinetics. Food Chem..

[31] Unnikrishnan P., Puthenveetil Kizhakkethil B., Annamalai J., Ninan G., Aliyamveetil Abubacker Z., Chandragiri Nagarajarao R. (2019). Tuna Red Meat Hydrolysate as Core and Wall Polymer for Fish Oil Encapsulation: A Comparative Analysis. J. Food Sci. Technol..

[32] Shahidi F. (2001). Headspace Volatile Aldehydes as Indicators of Lipid Oxidation in Foods. Headspace Analysis of Food and Flavors: Theory and Practice.

[33] Boerekamp D.M.W., Andersen M.L., Jacobsen C., Chronakis I.S., García-Moreno P.J. (2019). Oxygen Permeability and Oxidative Stability of Fish-oil-loaded Electrosprayed Capsules Measured by Electron Spin Resonance: Effect of Dextran and Glucose Syrup as Main Encapsulating Materials. Food Chem..

[34] Bakry A.M., Fang Z., Ni Y., Cheng H., Chen Y.Q., Liang L. (2016). Stability of Tuna Oil and Tuna Oil/Peppermint Oil Blend Microencapsulated Using Whey Protein Isolate in Combination with Carboxymethyl Cellulose or Pullulan. Food Hydrocoll..

[35] Xu L., Yu X., Li M., Chen J., Wang X. (2018). Monitoring Oxidative Stability and Changes in Key Volatile Compounds in Edible Oils during Ambient Storage through HS-SPME/GC–MS. Int. J. Food Prop..

[36] van Ruth S.M., Roozen J.P. (2000). Release of Volatile Oxidation Products from Sunflower Oil and Its Oil-in-Water Emulsion in a Model Mouth System. ACS Symp. Ser..

[37] Xiang J., Liu F., Wang B., CHen L., Liu W., Tan S. (2021). A Literature Review on Maillard Reaction Based on Milk Products: Advantages, Disadvantages and Avoidance Strategies. Foods.

[38] Carneiro H.C.F., Tonon R.V., Grosso C.R.F., Hubinger M.D. (2013). Encapsulation Efficiency and Oxidative Stability of Flaxseed Oil Microencapsulated by Spray Drying Using Different Combinations of Wall Materials. J. Food Eng..

[39] Henna Lu F.S., Nielsen N.S., Jacobsen C. (2013). Comparison of Two Methods for Extraction of Volatiles from Marine PL Emulsions. Eur. J. Lipid Sci. Technol..

