# Supplementary material for: Comparative Study on the Oxidative Stability of Encapsulated Fish Oil by Monoaxial or Coaxial Electrospraying and Spray-Drying
Source: Antioxidants (Basel). 2023 Jan 24;12(2):266. doi: 10.3390/antiox12020266 (PMC9952345; doi:10.3390/antiox12020266)
Supplement: Supplementary file 1 [file antioxidants-12-00266-s001.zip › antioxidants-2138387-supplementary.pdf]

SUPPLEMENTARY MATERIAL

(A) (B)

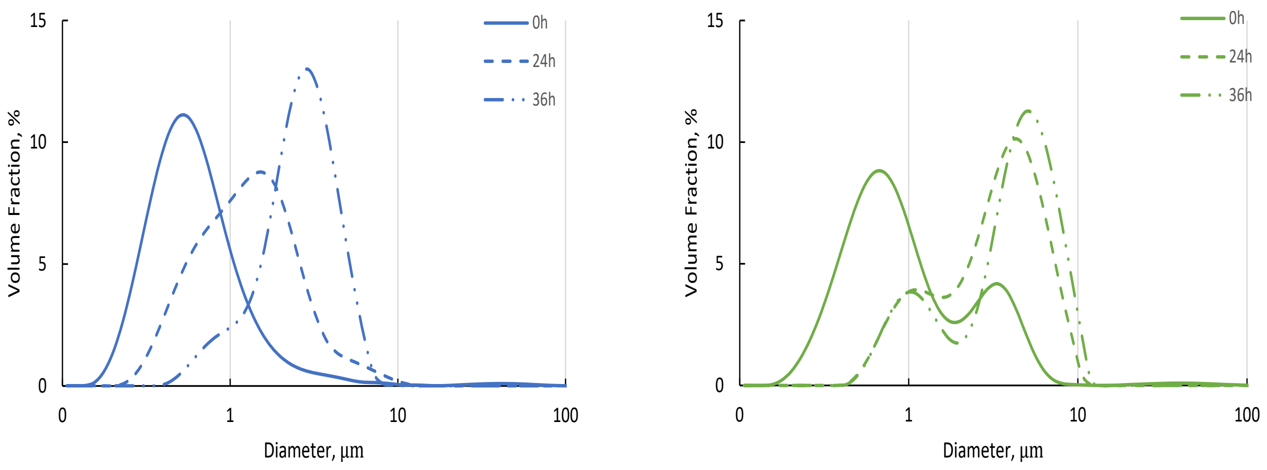

Figure S1. Oil droplet size distribution of the fish-oil-loaded emulsions produced for monoaxial electrospraying during processing (0h, 24h, 36h): GS-mo **(A)** and MD-mo **(B)**.

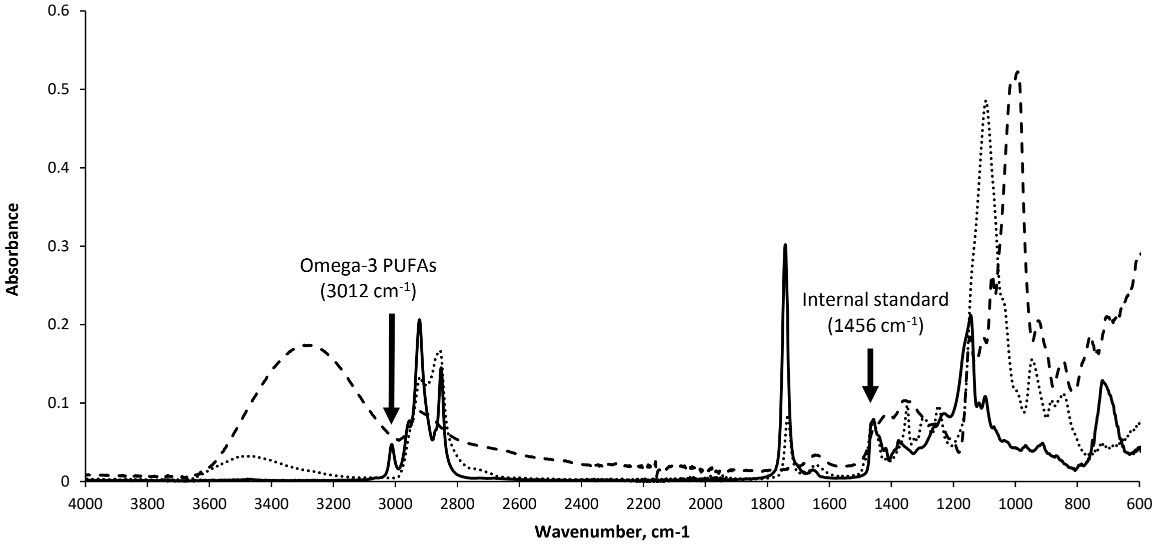

Figure S2. ATR-FTIR spectra of the: fish oil (solid line), glucose syrup (dashed line) and Tween-20 (dotted line).

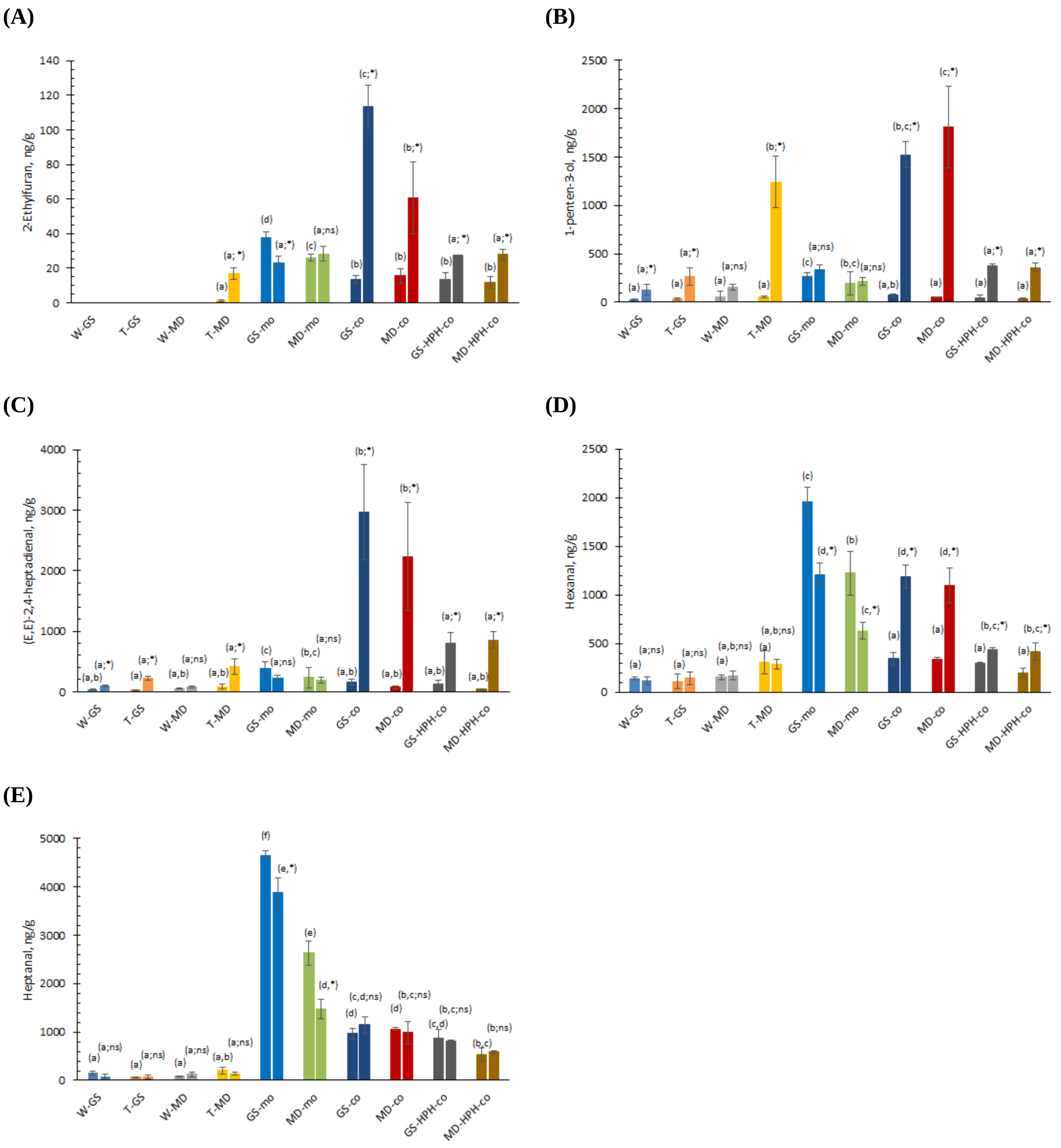

Figure S3. Secondary volatile oxidation products (SVOPs) of the fish-oil-loaded capsules produced by spray-drying or electrospraying (monoaxial and coaxial) at week 0 and week 6 of storage: 2-ethylfuran **(A)**; 1-penten-3-ol **(B)**; (*E*,*E*)-2,4-heptadienal **(C)**; hexanal **(D)**; heptanal **(E)**. Samples followed by a letter, a-d, indicates statistical differences ($p \leq 0.05$) between samples at the same sampling point. Samples followed by an asterisk (*) indicates statistical differences (p · 0.05) between week 0 and week 6. Means within the same sample followed by "ns" indicates no statistical differences ($p > 0.05$) between week 0 and week 6.
